# Direct Observation of Electrically Conductive Pili Emanating from *Geobacter sulfurreducens*

**DOI:** 10.1128/mBio.02209-21

**Published:** 2021-08-31

**Authors:** Xinying Liu, David J. F. Walker, Stephen S. Nonnenmann, Dezhi Sun, Derek R. Lovley

**Affiliations:** a Department of Microbiology, University of Massachusetts—Amherst, Amherst, Massachusetts, USA; b College of Environmental Science and Engineering, Beijing Forestry University, Beijing, China; c Department of Molecular Biosciences, University of Texas at Austin, Austin, Texas, USA; d Institute for Applied Life Sciences, university of Massachusetts—Amherst, Amherst, Massachusetts, USA; e Department of Mechanical and Industrial Engineering, University of Massachusetts—Amherst, Amherst, Massachusetts, USA; University of Delaware

**Keywords:** *Geobacter*, cytochromes, electromicrobiology, microbial nanowires, pili

## Abstract

Geobacter sulfurreducens is a model microbe for elucidating the mechanisms for extracellular electron transfer in several biogeochemical cycles, bioelectrochemical applications, and microbial metal corrosion. Multiple lines of evidence previously suggested that electrically conductive pili (e-pili) are an essential conduit for long-range extracellular electron transport in G. sulfurreducens. However, it has recently been reported that G. sulfurreducens does not express e-pili and that filaments comprised of multi-heme *c*-type cytochromes are responsible for long-range electron transport. This possibility was directly investigated by examining cells, rather than filament preparations, with atomic force microscopy. Approximately 90% of the filaments emanating from wild-type cells had a diameter (3 nm) and conductance consistent with previous reports of e-pili harvested from G. sulfurreducens or heterologously expressed in Escherichia coli from the G. sulfurreducens pilin gene. The remaining 10% of filaments had a morphology consistent with filaments comprised of the *c*-type cytochrome OmcS. A strain expressing a modified pilin gene designed to yield poorly conductive pili expressed 90% filaments with a 3-nm diameter, but greatly reduced conductance, further indicating that the 3-nm diameter conductive filaments in the wild-type strain were e-pili. A strain in which genes for five of the most abundant outer-surface *c*-type cytochromes, including OmcS, were deleted yielded only 3-nm-diameter filaments with the same conductance as in the wild type. These results demonstrate that e-pili are the most abundant conductive filaments expressed by G. sulfurreducens, consistent with previous functional studies demonstrating the need for e-pili for long-range extracellular electron transfer.

## OBSERVATION

Electroactive microorganisms are important in multiple biogeochemical cycles, the human gut, several bioenergy strategies, and metal corrosion (1, 2). One of the most contentious issues in electromicrobiology has been the role of electrically conductive protein nanowires in facilitating long-range electron transport. Electrically conductive protein nanowires have been studied most extensively in Geobacter sulfurreducens, which has served as the model microbe for elucidating the mechanisms of long-range electron transport in *Geobacter* species (3). *Geobacter* spp. are of interest because they are often the most abundant electroactive microbes in soils and sediments in which organic matter oxidation is coupled to Fe(III) oxide reduction, in natural methanogenic environments and anaerobic digesters where they serve as electron-donating partners for direct interspecies electron transfer (DIET) with methanogens, and in electrode biofilms harvesting electricity from waste organic matter (3–5). Furthermore, *Geobacter* are the most effective microbes available in culture for extracellular electron transport functions such as Fe(III) oxide reduction (3), producing electric current (5), DIET (6), and corrosion via direct extraction of electrons from metallic iron (7, 8). An additional area of interest is the potential for constructing electronic devices with novel functions with G. sulfurreducens protein nanowires (9).

Debate has arisen over the composition of G. sulfurreducens protein nanowires and their role in long-range electron transfer. Multiple lines of evidence have suggested that electrically conductive pili (e-pili) are the most abundant G. sulfurreducens protein nanowires and that e-pili are essential for long-range electron transport (10, 11). However, two recent publications have suggested that G. sulfurreducens does not express e-pili and that protein nanowires comprised of the multi-heme *c-*type cytochromes OmcS and OmcZ are the functional conduits for long-range extracellular electron transfer (12, 13). The primary argument against the production of e-pili is the fact filaments comprised of *c*-type cytochromes are the most abundant filaments observed in filament preparations observed with cryo-electron microscopy (12, 13). However, generating these filament preparations involves shearing filaments from the cell, purifying the filaments under high pH, selective precipitation with ammonium sulfate, and affixing filaments to grids. Each of these steps has the potential to selectively enrich specific filaments or for artifactual formation of cytochrome filaments (11).

## DIRECT EXAMINATION OF CELLS WITH ATOMIC FORCE MICROSCOPY

In order to avoid potential artifacts/enrichments associated with filament purification, the filaments associated with G. sulfurreducens cells were directly examined with atomic force microscopy (AFM). Cells were grown in medium with acetate (10 mM) as electron donor and fumarate (40 mM) as electron acceptor as previously described (14), a growth condition in which expression of OmcS is expected to be high (12, 15, 16). An aliquot (50 μl) of culture was drop cast onto a silicon wafer coated with a 35 nm layer of platinum, prepared as previously described (17). After 12 min, excess liquid was removed with a pipette, and the substrate was washed twice with 50 μl of deionized water. Excess water was absorbed with filter paper, the preparation was allowed to air dry, and samples were equilibrated at 40% humidity inside the scanning chamber of the atomic force microscope prior to examination.

**FIG 1:**
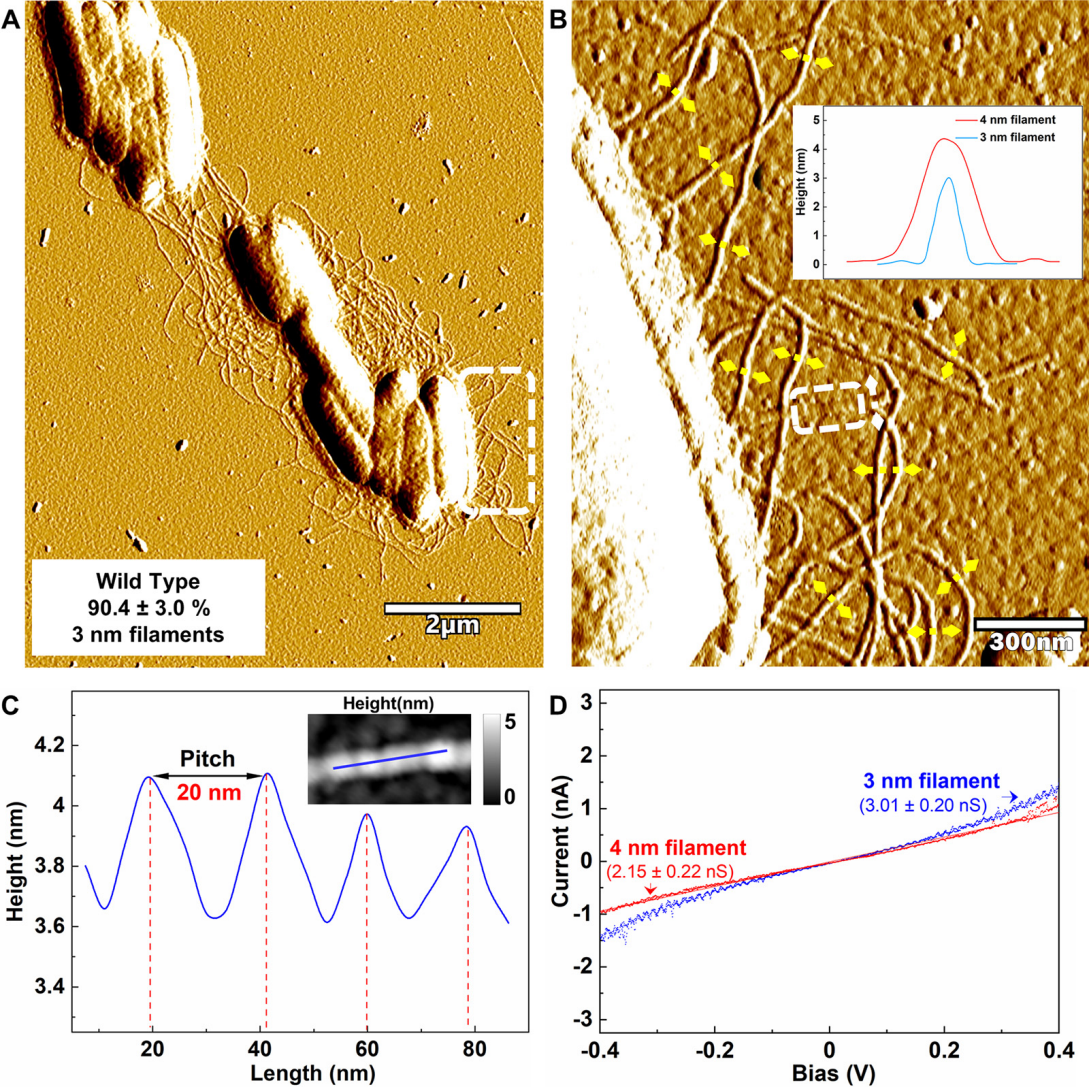
Characterization of filaments emanating from G. sulfurreducens with the wild-type pilin gene. (A) AFM amplitude image. The proportion of 3-nm-diameter filaments was calculated from the total number of 3- and 4-nm-diameter filaments counted in nine regions from three separate samples (see Fig. S2 and S3) and were determined from height images similar to those shown in Fig. S1 (see Table S1 for statistical analysis and Data Set S1 for all raw data). (B) Higher magnification of the region highlighted in the dashed frame in panel A. The inset shows typical height profiles across the 3-nm (yellow lines)- and 4-nm (white line)-diameter filaments, as determined from the corresponding height images (see Fig. S1B). Due to fluctuation of diameter along the axis of the filaments, diameters were measured at the points of greatest diameter for consistency. (C) Longitudinal height profile (along solid blue line in inset) for region on the 4-nm filament noted by the white dashed frame in panel B. (D) Comparison of point-mode current response (I-V) spectroscopy for 4-nm (red)- and 3-nm (blue)-diameter filaments. The responses shown are representative of three different measurements on each of three individual filaments (see Fig. S4). Conductance (mean plus the standard deviation, *n* = 9) was calculated from a linear fit model between −0.2 V and 0.2 V (see Fig. S4).

AFM (see Text S1 in the supplemental material for detailed methods) revealed cells with abundant filaments (Fig. 1A; see also Fig. S1A). There were two types of filaments emanating from the cells. One filament type appeared to be comprised of OmcS, as evidenced from its 4 nm diameter (Fig. 1B; see also Fig. S1B) and its characteristic axial periodicity with a 20-nm pitch (12, 18) (Fig. 1C). The OmcS filaments consistently accounted for only ca. 10% of the filaments observed (Fig. 1A; see also Fig. S2 and S3 and Table S1).

10.1128/mBio.02209-21.1 DATA SET S1 Original data for filament diameter measurements. Download Data Set S1, XLSX file, 0.2 MB. Copyright © 2021 Liu et al. 2021 Liu et al. https://creativecommons.org/licenses/by/4.0/ This content is distributed under the terms of the Creative Commons Attribution 4.0 International license.

**FIG 2:**
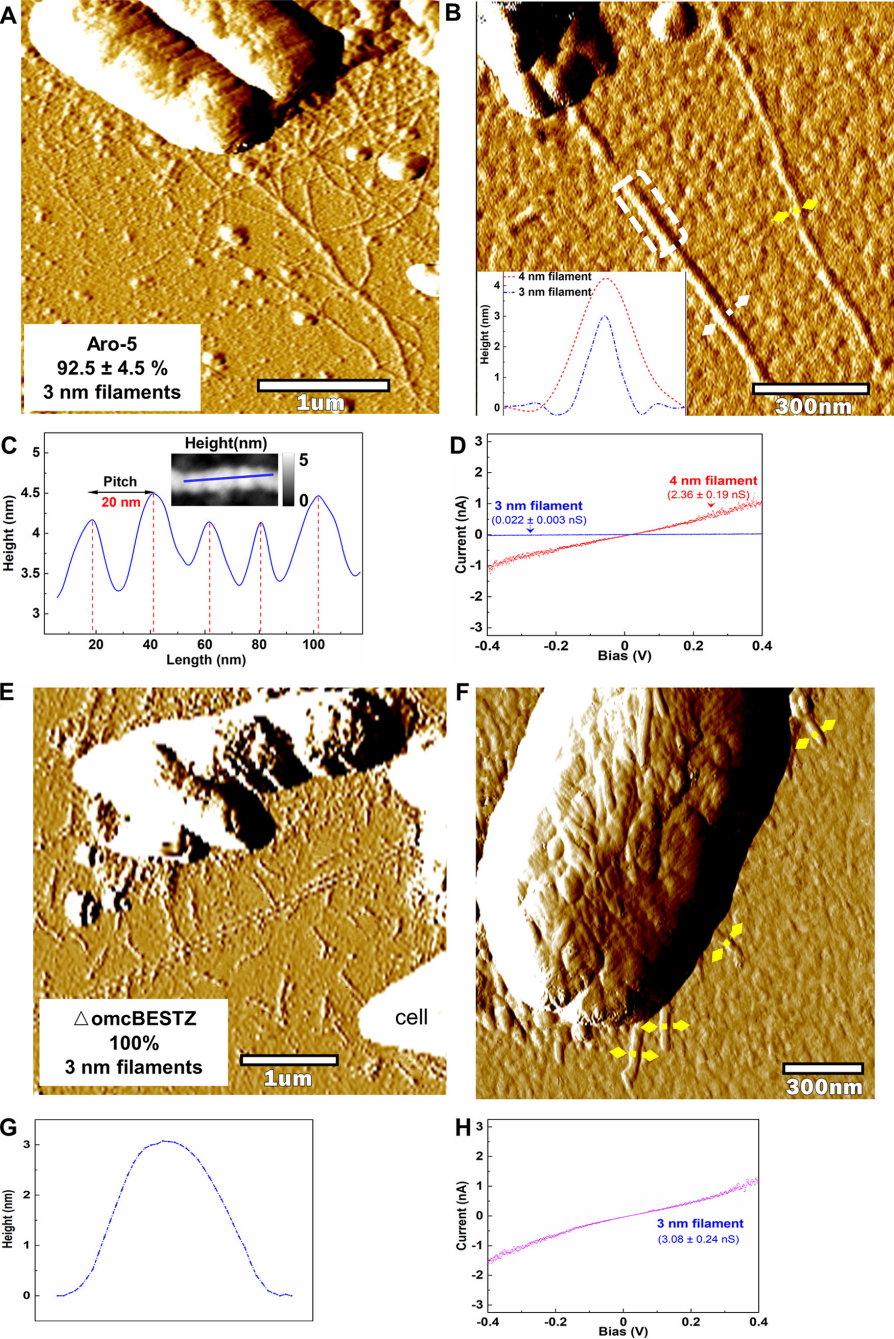
Characterization of filaments emanating from G. sulfurreducens strain Aro-5 and strain △omcBESTZ. (A) AFM amplitude image of filaments associated with strain Aro-5. The proportion of 3-nm-diameter filaments was calculated from the total number of 3-nm and 4-nm-diameter filaments counted in six regions from three separate samples (see Fig. S5) and were determined from height images similar to those shown in Fig. 1 (see Table S1 for statistical analysis and Data Set S1 for all raw data). (B) AFM amplitude image at higher magnification illustrating the two filament types. Inset shows typical height profiles across the 3-nm (yellow lines)- and 4-nm (white line)-diameter filaments, as determined from the corresponding height images (see Fig. S1D). (C) Longitudinal height profile (along solid blue line in inset) for the portion of the 4-nm-diameter filament within the white frame in panel B. (D) Comparison of point-mode current response (I-V) spectroscopy for 4-nm (red) and 3-nm (blue) filaments. The responses shown are representative of three different measurements on three individual wires (see Fig. S6). Conductance (mean plus the standard deviation, *n* = 9) was calculated from a linear fit model between −0.2 V and 0.2 V (see Fig. S6). (E) AFM amplitude image of filaments associated with strain △omcBESTZ. (F) AFM amplitude image at higher magnification showing 3-nm-diameter filaments emanating from cell of strain △omcBESTZ. (G) Typical height profile across the filaments designated by yellow lines in panel F, as determined from the corresponding height images (see Fig. S1F, Table S1, and Data Set S1). (H) Point-mode current response (I-V) spectroscopy representative of three different measurements on three individual wires (see Fig. S7) on 3 nm filaments emanating from strain △omcBESTZ. Conductance (mean + standard deviation, *n* = 9) was calculated from a linear fit model between −0.2 V and 0.2 V (see Fig. S7).

10.1128/mBio.02209-21.3 FIG S1 AFM height images corresponding to amplitude images of the primary text. (A) AFM height image of filaments emanating from G. sulfurreducens with the wild-type pilin gene (corresponding to Fig. 1A). (B) AFM height image of higher magnification of the region highlighted in the dashed frame in panel A (corresponding to Fig. 1B). (C) AFM height image of filaments emanating from G. sulfurreducens strain Aro-5 (corresponding to Fig. 2A). (D) AFM height image of strain Aro-5 at higher magnification illustrating the two filament types with yellow and white dashed lines designating cross sections for the 3-nm and 4-nm-diameter filaments, respectively (corresponding to Fig. 2B). (E) AFM height image of filaments emanating from G. sulfurreducens strain △omcBESTZ (corresponding to Fig. 2E). (F) AFM height image at higher magnification showing 3-nm-diameter filaments emanating from cell of the *ΔomcBESTZ* strain (corresponding to Fig. 2F). All height profiles diameter measurements are reported in Data Set S1. Download FIG S1, TIF file, 2.9 MB. Copyright © 2021 Liu et al. 2021 Liu et al. https://creativecommons.org/licenses/by/4.0/ This content is distributed under the terms of the Creative Commons Attribution 4.0 International license.

10.1128/mBio.02209-21.2 TEXT S1 Atomic force microscopy analysis. Download Text S1, DOCX file, 0.02 MB. Copyright © 2021 Liu et al. 2021 Liu et al. https://creativecommons.org/licenses/by/4.0/ This content is distributed under the terms of the Creative Commons Attribution 4.0 International license.

10.1128/mBio.02209-21.4 FIG S2 AFM amplitude image of filaments emanating from G. sulfurreducens with the wild-type pilin gene in sample 1, designating the six regions used to count the number of 3- and 4-nm-diameter filaments. Height profiles were determined from the corresponding height images, which yield profiles similar to those shown in Fig. S1. Download FIG S2, TIF file, 1.4 MB. Copyright © 2021 Liu et al. 2021 Liu et al. https://creativecommons.org/licenses/by/4.0/ This content is distributed under the terms of the Creative Commons Attribution 4.0 International license.

10.1128/mBio.02209-21.5 FIG S3 (A) Higher magnification of five regions noted in Fig. S2. Crosses designate the counted 3 nm (blue) and 4 nm (red) diameter filaments. (B) AFM amplitude image of filaments emanating from G. sulfurreducens with the wild-type pilin gene in sample 2 (designated regions VI and VII) and sample 3 (designated regions VIII and IX). Crosses designate the counted 3-nm (blue)- and 4-nm (red)-diameter filaments. Height profiles were determined from the corresponding height images, which yield profiles similar to those shown in Fig. S1. Insets show the results for each section. Supporting data are provided in Table S1 and Data Set S1. Download FIG S3, TIF file, 2.0 MB. Copyright © 2021 Liu et al. 2021 Liu et al. https://creativecommons.org/licenses/by/4.0/ This content is distributed under the terms of the Creative Commons Attribution 4.0 International license.

10.1128/mBio.02209-21.10 TABLE S1 Statistics for filaments emanating from the three different strains of G. sulfurreducens examined. Download Table S1, DOCX file, 0.02 MB. Copyright © 2021 Liu et al. 2021 Liu et al. https://creativecommons.org/licenses/by/4.0/ This content is distributed under the terms of the Creative Commons Attribution 4.0 International license.

10.1128/mBio.02209-21.6 FIG S4 (A) Point-mode current response (I-V) spectroscopy measurements and conductance calculations of the three independent 3 nm diameter filaments from G. sulfurreducens with the wild-type pilin gene shown in Fig. 1D of the primary text. Three independent filaments are shown as rows a, b, and c. Three independent measurements on each filament are shown as columns 1, 2, and 3. (B) Point-mode current response (I-V) spectroscopy measurements and conductance calculations of the three independent 4-nm-diameter filaments from G. sulfurreducens with the wild-type pilin gene shown in Fig. 1D of the primary text. Three independent filaments are shown as rows a, b, and c. Three independent measurements on each filament are shown as column 1, 2 and 3. Download FIG S4, TIF file, 1.1 MB. Copyright © 2021 Liu et al. 2021 Liu et al. https://creativecommons.org/licenses/by/4.0/ This content is distributed under the terms of the Creative Commons Attribution 4.0 International license.

Approximately 90% of the filaments were 3 nm in diameter (Fig. 1A; see also Fig. S2 and S3 and Table S1), the same diameter as the filaments observed when the G. sulfurreducens PilA pilin gene is expressed in Pseudomonas aeruginosa (19) or Escherichia coli (20) and the same diameter of individual conductive filaments previously harvested from G. sulfurreducens (21, 22). These results suggest that the 3-nm-diameter filaments are e-pili. As expected from the growth conditions used, no filaments with a morphology consistent with the 2.5-nm diameter and axial pitch of OmcZ filaments (13) were observed. Both the OmcS and e-pili filaments exhibited an ohmic-like response (Fig. 1D; see also Fig. S4). The conductance of the e-pili was slightly higher than that of the OmcS filaments (Fig. 1D; see also Fig. S4).

G. sulfurreducens strain Aro-5 was previously constructed to replace the PilA pilin gene with *aro-5*, a synthetic pilin gene designed to yield poorly conductive pili (23). The conductivity of filaments harvested from the cells is much lower than the conductivity of filaments harvested from wild-type controls (22–25). Direct examination of filaments emanating from strain Aro-5 revealed two types of filaments, morphologically similar to those observed in the wild-type control (Fig. 2A and B; see also Fig. S1C and D). Filaments with a diameter and longitudinal pitch (Fig. 2C) consistent with OmcS filaments comprised ca. 10% of the filaments (Fig. 2A; see also Fig. S5 and Table S1), similar to the OmcS filament abundance in the wild-type control and consistent with the observation that strain Aro-5 produces abundant OmcS (23). The conductance of these 4-nm-diameter filaments was the same as the conductance observed for the OmcS filaments of the wild-type control (Fig. 2D; see also Fig. S6A). As with the wild-type strain, the 3 nm diameter filaments accounted for ca. 90% of the filaments observed, but their conductance was more than 100-fold lower (Fig. 2D; see also Fig. S6B). This decreased conductance is in agreement with previous observations of attenuated conductivity in filaments harvested from strain Aro-5, including measurements on individual 3-nm-diameter filaments (22–25). The dramatic change in the conductance of the 3-nm filaments emanating from cells associated with the expression of *aro-5* pilin gene provides further evidence that the 3-nm filaments in the wild-type strain were e-pili.

10.1128/mBio.02209-21.7 FIG S5 (A) AFM amplitude image of filaments emanating from G. sulfurreducens strain Aro-5 in sample 1 (regions I and II) and sample 2 (regions III and IV). Crosses designate the counted 3-nm (blue)- and 4-nm (red)-diameter filaments. (B) AFM amplitude image of filaments emanating from G. sulfurreducens strain Aro-5 in sample 3, regions V and VI. Crosses designate the counted 3-nm (blue)- and 4-nm (red)-diameter filaments. Height profiles were determined from the corresponding height images, which yield profiles similar to those shown in Fig. S1. Insets show the results for each section. Supporting data are provided in Table S1 and Data Set S1. Download FIG S5, TIF file, 2.1 MB. Copyright © 2021 Liu et al. 2021 Liu et al. https://creativecommons.org/licenses/by/4.0/ This content is distributed under the terms of the Creative Commons Attribution 4.0 International license.

10.1128/mBio.02209-21.8 FIG S6 (A) Point-mode current response (I-V) spectroscopy measurements and conductance calculations of the three independent 4-nm-diameter filaments from G. sulfurreducens strain Aro-5 shown in Fig. 2D of the primary text. Three independent filaments are shown as rows a, b, and c. Three independent measurements on each filament are shown as columns 1, 2, and 3. (B) Point-mode current response (I-V) spectroscopy measurements and conductance calculations of the three independent 3-nm-diameter filaments from G. sulfurreducens strain Aro-5 shown in Fig. 2D of the primary text. Three independent filaments are shown as rows a, b, and c. Three independent measurements on each filament are shown as columns 1, 2, and 3. Download FIG S6, TIF file, 1.0 MB. Copyright © 2021 Liu et al. 2021 Liu et al. https://creativecommons.org/licenses/by/4.0/ This content is distributed under the terms of the Creative Commons Attribution 4.0 International license.

10.1128/mBio.02209-21.9 FIG S7 Point-mode current response (I-V) spectroscopy measurements and conductance calculations of the three independent 3-nm-diameter filaments from the G. sulfurreducens Δ*omcBESTZ* strain shown in Fig. 2F in the primary text. Three independent filaments are shown as row a, b, and c. Three independent measurements on each filament are shown as columns 1, 2, and 3. Download FIG S7, TIF file, 0.6 MB. Copyright © 2021 Liu et al. 2021 Liu et al. https://creativecommons.org/licenses/by/4.0/ This content is distributed under the terms of the Creative Commons Attribution 4.0 International license.

In order to further investigate the possibility of cytochrome-based filaments, we next examined the previously described Δ*omcBESTZ* strain (26) in which the genes for the most abundant G. sulfurreducens outer surface multi-heme *c*-type cytochromes—OmcB, OmcE, OmcS, OmcT, and OmcZ—were deleted. As expected, filaments with morphologies consistent with OmcS-based filaments were not apparent in this strain. All of the filaments emanating from the Δ*omcBESTZ* strain and lying near the cells were short, but had a diameter of 3 nm (Fig. 2E and F; see also Fig. S1E and F). Their conductance was the same as for the 3-nm filaments of the wild-type strain (Fig. 2H; see also Fig. S7).

## IMPLICATIONS

The results of direct observation of filaments emanating from cells of G. sulfurreducens demonstrates that G. sulfurreducens copiously expresses filaments with properties expected for e-pili. The e-pili were ∼10-fold more abundant than putative OmcS filaments. These observations are in accordance with a number of previous observations. For example, when a pilin monomer modified with a peptide tag was expressed in G. sulfurreducens, all of the filaments observed emanating from the cells were also decorated with the peptide tag (27). Several studies reported recovery of electrically conductive 3-nm-diameter filaments when filaments were sheared off the outer surface of G. sulfurreducens (21, 22, 27) or when the G. sulfurreducens pilin monomer was expressed in Pseudomonas aeruginosa (19) or Escherichia coli (20). Furthermore, as shown here, expressing *aro-5* instead of the wild-type *pilA* resulted in 3-nm filaments emanating from the cells with a similar morphology, but greatly attenuated conductance. Heterologously expressing a pilin gene encoding increased aromatic amino acid content yielded 3-nm-diameter filaments with 5,000-fold-higher conductivity than the wild-type control (28). These results are consistent with the expression of e-pili and inconsistent with cytochrome-based filaments, as was the finding reported here that the 3-nm filaments were still produced in a strain in which the genes for the most abundant outer-surface cytochromes were deleted. The abundance of e-pili in G. sulfurreducens is also consistent with the finding that microbes that do not express outer-surface *c*-type cytochromes can construct conductive filaments from monomers homologous to the G. sulfurreducens pilin monomer (24, 25, 29).

Notably, G. sulfurreducens strains that express pili of low conductance are consistently deficient in long-range extracellular electron transfer (23, 24, 30), providing strong evidence for the role of e-pili in extracellular electron transport. The same cannot be said of the cytochrome filaments OmcS and OmcZ. G. sulfurreducens strain Aro-5 cannot produce highly conductive biofilms or high current densities on anodes (23), despite, as shown here, continued capability to express OmcS filaments. Furthermore, deleting *omcS* has no negative impact on biofilm conductivity or current production (15, 31). Deletion of *omcS* can inhibit Fe(III) oxide reduction, in some, but not all variants of G. sulfurreducens (32, 33). When deletion of *omcS* does have an impact, the strain can be rescued for Fe(III) oxide reduction with the addition of ultrafine-grained magnetite (34). However, magnetite cannot substitute for e-pili, demonstrating an essential role for e-pili, but not OmcS, in Fe(III) oxide reduction. When considering the potential function of OmcS in extracellular electron transfer it is also important to note that OmcS attached to the cell surface, not in filamentous form, may predominate, especially in actively growing cells (16).

OmcZ is not required for Fe(III) oxide reduction (15) and is not highly expressed in cells reducing Fe(III) oxide (32). Although it was suggested that OmcZ filaments might account for the high conductivity of anode biofilms (13), this hypothesis is inconsistent with the poor current production by strain Aro-5 and the low conductivity of its biofilms (23). Furthermore, OmcZ is localized near the anode-biofilm interface, OmcZ filaments are not observed coursing through the bulk of the biofilm (35), and there is no correlation between OmcZ abundance and biofilm conductivity (31).

In conclusion, eliminating artifacts by directly examining filaments emanating from cells has demonstrated that G. sulfurreducens expresses e-pili in abundance, consistent with multiple lines of evidence from previous studies (10, 11) that have indicated that G. sulfurreducens e-pili are an important component in long-range extracellular electron transport. The cells examined produced few OmcS-based filaments. The physiological significance of cytochrome-based filaments is yet to be determined.
