# Supplementary figures and images for: Direct Observation of Electrically Conductive Pili Emanating from Geobacter sulfurreducens
Source: mBio. 2021 Aug 31;12(4):e02209-21. doi: 10.1128/mBio.02209-21 (PMC8406130; doi:10.1128/mBio.02209-21)

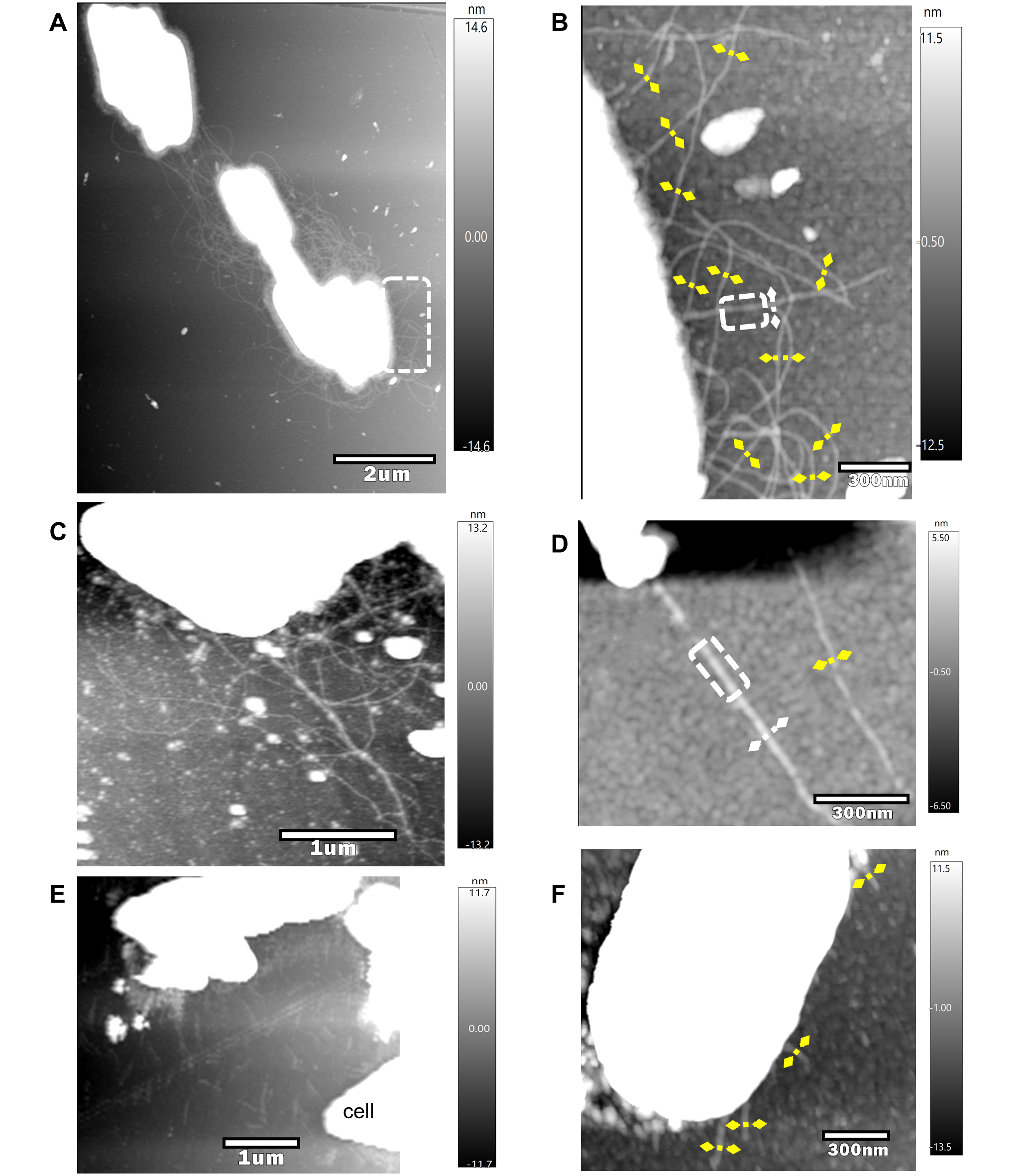

Supplement: FIG S1 [file mbio.02209-21-sf001.tif]

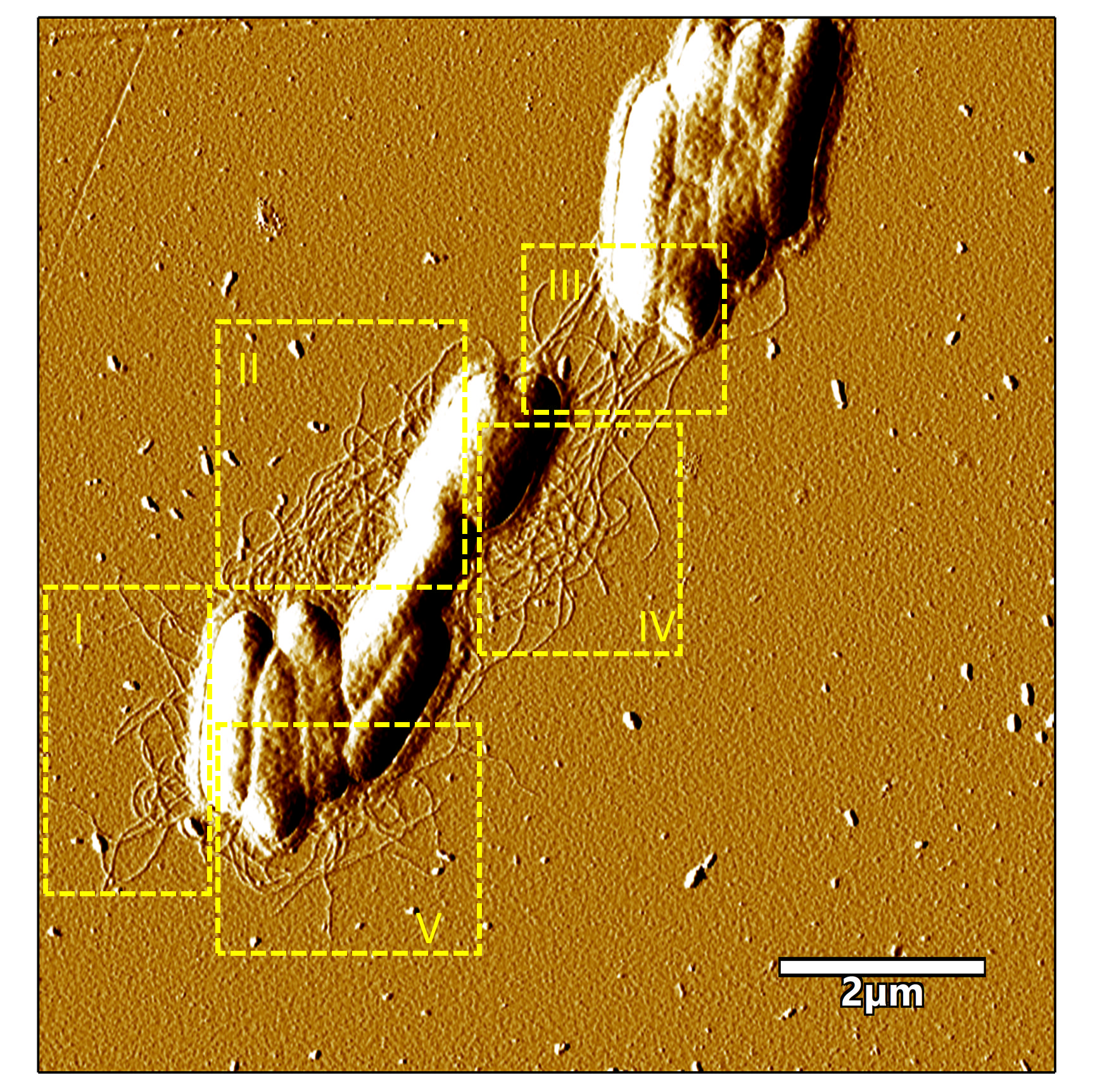

Supplement: FIG S2 [file mbio.02209-21-sf002.tif]

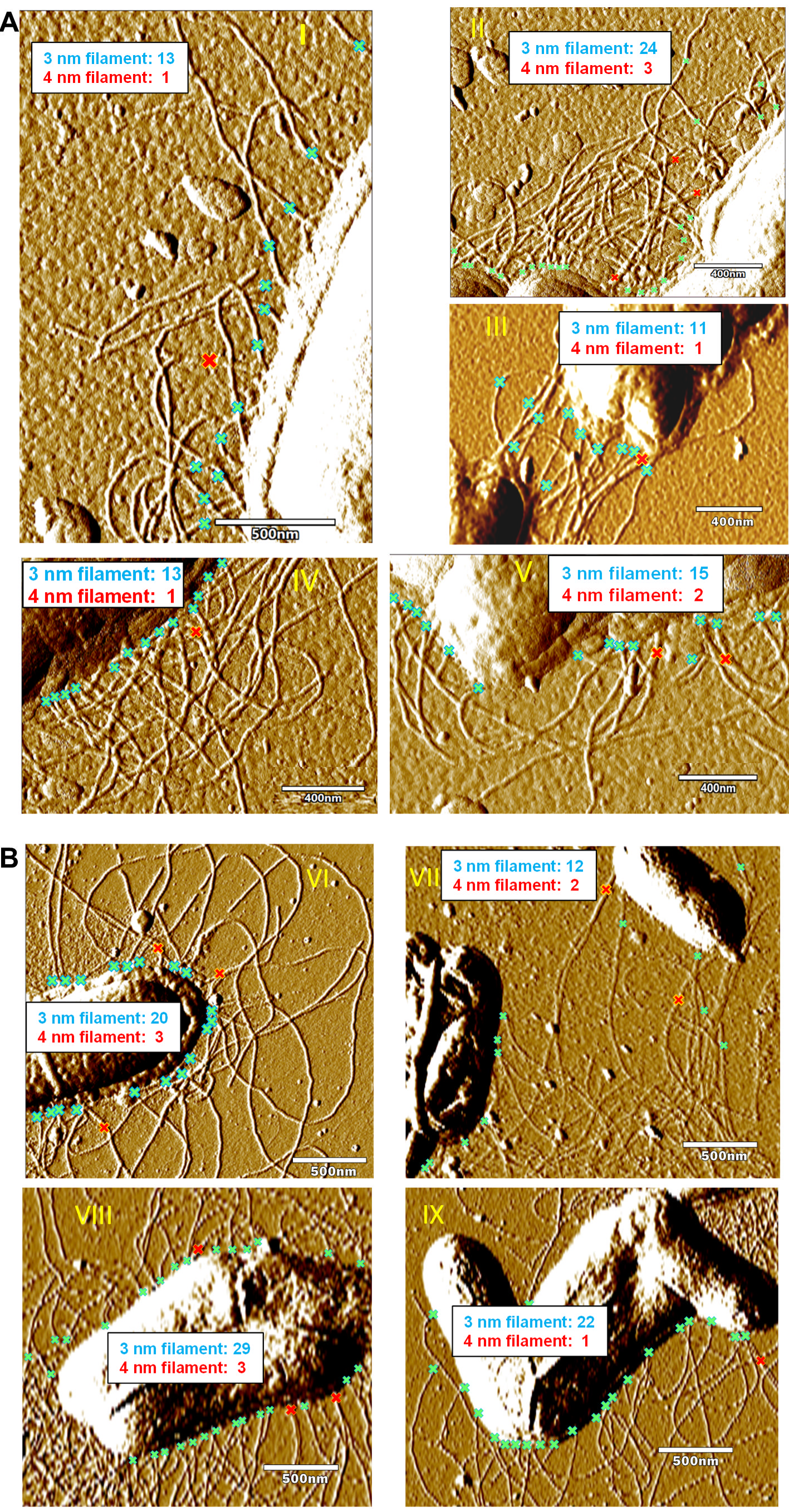

Supplement: FIG S3 [file mbio.02209-21-sf003.tif]

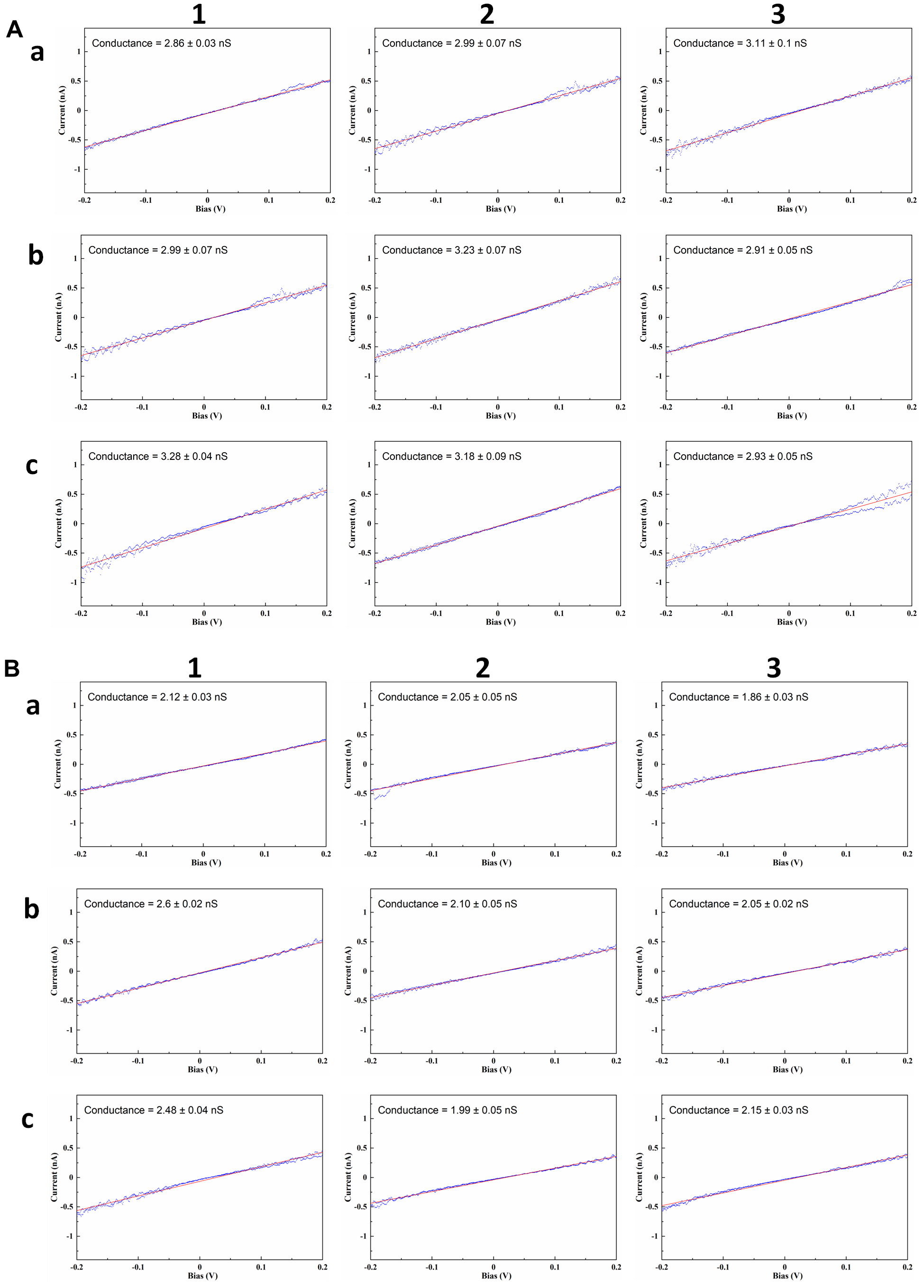

Supplement: FIG S4 [file mbio.02209-21-sf004.tif]

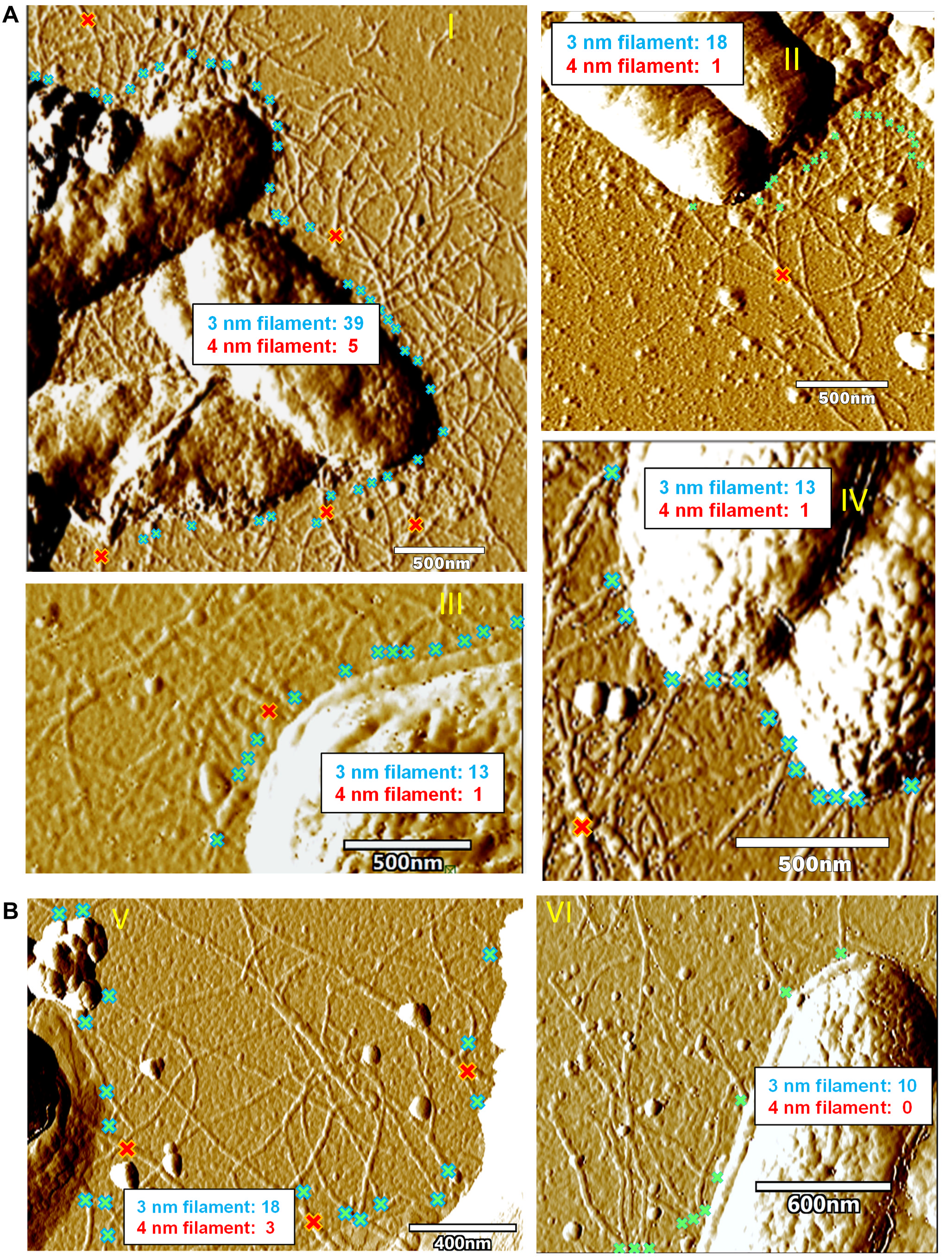

Supplement: FIG S5 [file mbio.02209-21-sf005.tif]

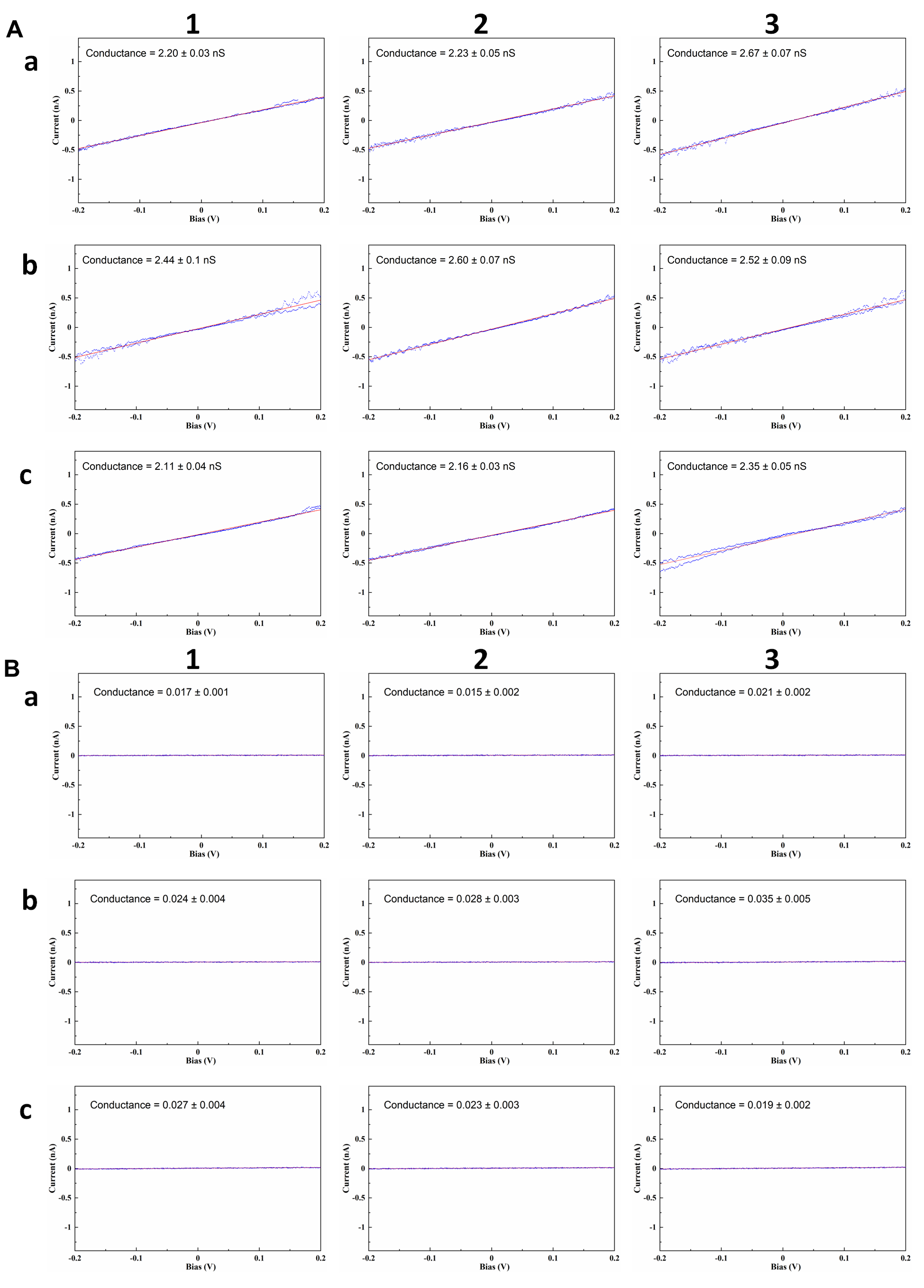

Supplement: FIG S6 [file mbio.02209-21-sf006.tif]

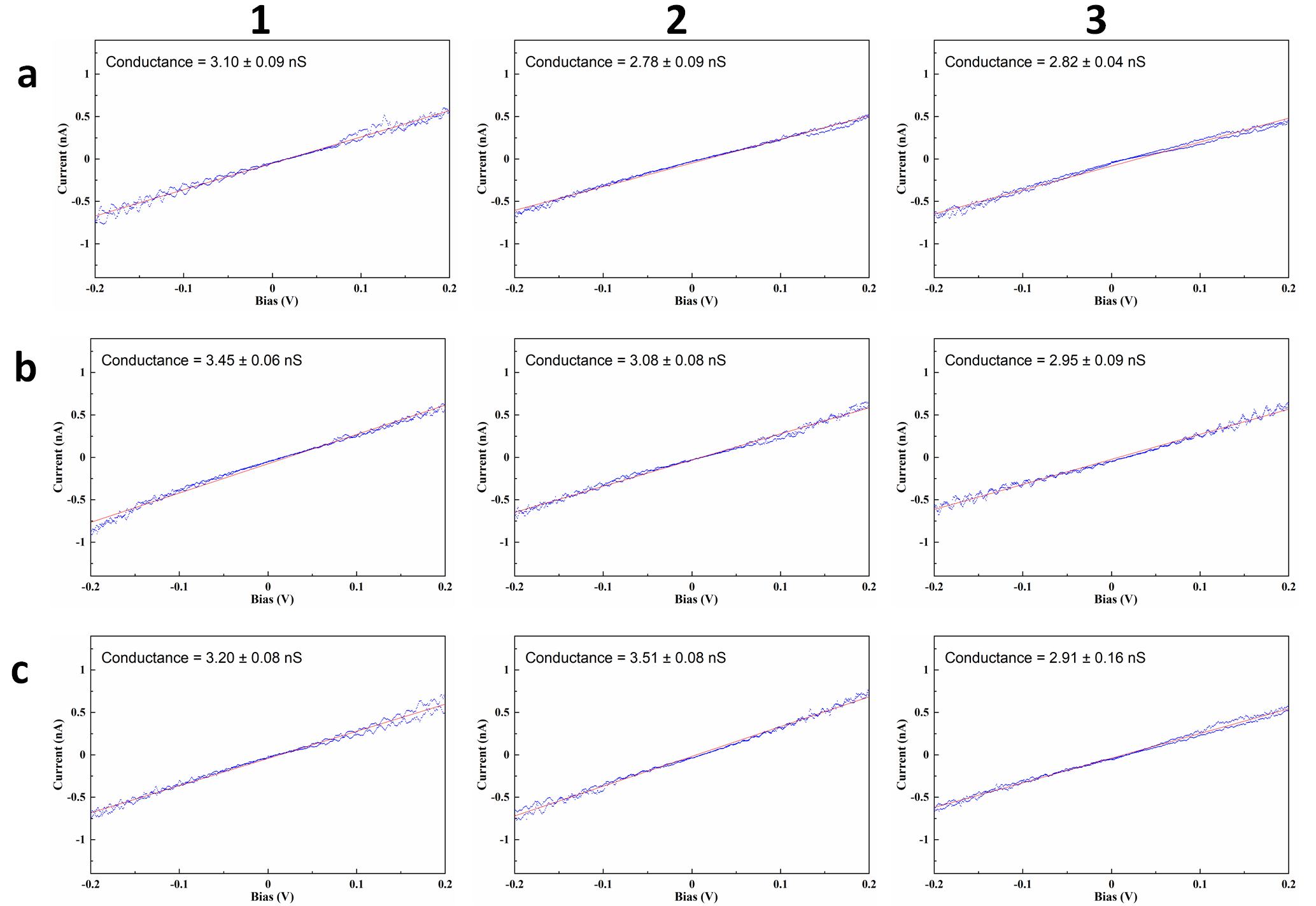

Supplement: FIG S7 [file mbio.02209-21-sf007.tif]
