# Supplementary material for: Direct Observation of Electrically Conductive Pili Emanating from Geobacter sulfurreducens
Source: mBio. 2021 Aug 31;12(4):e02209-21. doi: 10.1128/mBio.02209-21 (PMC8406130; doi:10.1128/mBio.02209-21)
Supplement: TEXT S1 [file mbio.02209-21-s0001.docx]

**Supplemental Text**

**Direct Observation of Electrically Conductive Pili Emanating from *Geobacter sulfurreducens***

Xinying Liu^a,b^, David J. F. Walker^c^, Stephen S. Nonnenmann^d,e^, Dezhi Sun^b^, Derek R. Lovley^a,d^

^a^ Department of Microbiology, University of Massachusetts—Amherst, Amherst, Massachusetts, USA

^b^ College of Environmental Science and Engineering, Beijing Forestry University, Beijing, 100083, China

^c^ Institute for Cellular and Molecular Biology, University of Texas at Austin, Austin, Texas 78712, USA

^d^ Institute for Applied Life Sciences, university of Massachusetts—Amherst, Amherst, Massachusetts, USA

^e^ Department of Mechanical and Industrial Engineering, University of Massachusetts—Amherst, Amherst, Massachusetts, USA

**Atomic Force Microscopy Analysis**

Silicon wafers were coated with a 35 nm layer of platinum as previously described (1). A 50 µl aliquot of cultures was drop cast onto wafers. After 12 min, excess liquid was removed with a pipette and the substrate was washed twice with 50 µl of deionized water. Excess water was absorbed with filter paper and the preparation was allowed to air dry. Samples were equilibrated at 40% humidity inside scanning chamber of a Cypher ES, atomic force microscope (Asylum Research, Oxford Instrument) for at least 1 h at 25 °C. The filaments were first observed with tapping mode (AC-air topography) under repulsive force with a Pt/Ir-coated tip (PtSi-FM, NanoWorld AG) at a ~2.0 N/m spring force constant and ~70 kHz resonance frequency.

Height profiles of cross-section lines of filaments were examined in AFM height images (raw data provided in the Extended Data file with horizontal and vertical data from cross sections desginated as ‘x (m)’ and ‘y (m)’, respectively). The filament diameter was determined from line graphs with ‘x (m)’ as the x axis and ‘y (m)’ as the y axis, from the height difference between the highest and lowest points of the curve along one down direction. Due to the fluctuation of the diameter along the axis of the filaments, all diameters were determined at the points of greatest diameter for consistency. Diameter range from 2.7 to 3.3 nm were counted as ‘3 nm’ filaments and range from 3.8 to 4.4 nm were counted as ‘4nm’ filaments. ImageJ software (https://imagej.net/Welcome) was used to count the number of each filament type.

The conductance of individual filaments was determined in contact mode (force 30 nN) with the Pt/Ir-coated tip functioning as the translatable top electrode. Quadruplicate amplitude of ±0.4 V voltage at 0.99 Hz frequency was applied to obtain ca. 8000 points per measurement. Three independent points from three individual filaments (biological replicates) were analyzed to determine the conductance. Conductance was calculated from the linear slope between -0.2 to 0.2 V followed with the equation: Conductance = Current/Voltage as preciously described (2).

**References Cited**

1. Zhou Z, López-Domínguez P, Abdullah M, Barber DM, Meng X, Park J, Van Driessche I, Schiffman JD, Crosby AJ, Kittilstved KR, Nonnenmann SS. 2021. Memristive behavior of mixed xxide nanocrystal assemblies. ACS Applied Materials & Interfaces 13:21635-21644.

2. Walker DJF, Martz E, Holmes DE, Zhou Z, Nonnenmann SS, Lovley DR. 2019. The archaellum of Methanospirillum hungatei is electrically conductive. mBio 10:e00579-19.
