# Supplementary material for: Direct Observation of Electrically Conductive Pili Emanating from Geobacter sulfurreducens
Source: mBio. 2021 Aug 31;12(4):e02209-21. doi: 10.1128/mBio.02209-21 (PMC8406130; doi:10.1128/mBio.02209-21)
Supplement: TABLE S1 [file mbio.02209-21-st001.docx]

**Supplemental Table 1.** Statistics for filaments emanating from the three different strains of *G. sulfurreducens* examined.

| Cell Type  & Region# | Filament Diameter^1^ & Counts | | | | % 3 nm  Diameter | % 3 nm Diameter for Cell Type^2^ |
| --- | --- | --- | --- | --- | --- | --- |
|  | 3 nm filaments | | 4 nm filaments | |  |  |
|  | Height (nm) | n^3^ | Height (nm) | n^3^ |  |  |
| WT^4^-I | 3.02±0.11 | 13 | 4.14^5^ | 1 | 92.9 |  |
| WT-II | 3.02±0.18 | 24 | 4.28±0.12 | 3 | 88.9 |  |
| WT-III | 3.00±0.12 | 11 | 4.09^5^ | 1 | 91.7 |  |
| WT-IV | 3.03±0.14 | 13 | 4.24^5^ | 1 | 92.9 |  |
| WT-V | 3.02±0.11 | 15 | 4.28^5^ | 2 | 88.2 |  |
| WT-VI | 2.97±0.12 | 20 | 4.26±0.23 | 3 | 87.0 |  |
| WT-VII | 2.96±0.13 | 12 | 4.09^5^ | 2 | 85.7 |  |
| WT-VIII | 2.99±0.15 | 29 | 4.20±0.17 | 3 | 90.6 |  |
| WT-IX | 2.95±0.12 | 22 | 4.14^5^ | 1 | 95.7 |  |
| Total WT | 3.00±0.14 | 159 | 4.21±0.15 | 17 |  | 90.4 ± 3.0 |
|  |  |  |  |  |  |  |
| Aro5^6^-I | 2.97±0.16 | 39 | 4.11±0.11 | 5 | 88.6 |  |
| Aro5-II | 2.95±0.15 | 18 | 4.18^5^ | 1 | 94.7 |  |
| Aro5-III | 2.93±0.16 | 13 | 4.27^5^ | 1 | 92.9 |  |
| Aro5-IV | 2.95±0.15 | 13 | 4.37^5^ | 1 | 92.9 |  |
| Aro5-V | 2.93±0.15 | 18 | 4.14±0.08 | 3 | 85.7 |  |
| Aro5-VI | 2.90±0.15 | 10 | - | 0 | 100 |  |
| Total Aro-5 | 2.95±0.16 | 111 | 4.16±0.11 | 11 |  | 92.5 ± 4.5 |
|  |  |  |  |  |  |  |
| ∆BESTZ^7^-I | 2.94±0.14 | 15 | - | 0 | 100 |  |
| ∆BESTZ-II | 3.03±0.10 | 5 | - | 0 | 100 |  |
| Total ∆BESTZ | 2.96±0.14 | 20 | - | 0 | 100 | 100 |

^1^Mean + standard deviation

^2^Mean + standard deviation for cell type

^3^Number of filaments counted

^4^WT-strain expressing wild-type pilin gene

^5^Only show mean value is shown when there were not sufficient filaments measured to determine a standard deviation.

^6^Strain Aro5, which expresses a synthetic pilin gene designed to yield poorly conductive pili

^7^Strain ∆BESTZ in which the genes for five abundant outer-surface *c*-type cytochromes (OmcB, OmcE, OmcS, OmcT, and OmcZ) were deleted
